# The Temperature-Dependent Tight Binding Theory Modelling of Strain and Composition Effects on the Electronic Structure of CdSe- and ZnSe-Based Core/Shell Quantum Dots

**DOI:** 10.3390/ma18020283

**Published:** 2025-01-10

**Authors:** Derya Malkoç, Hilmi Ünlü

**Affiliations:** 1 Nanoscience and Nanoengineering Programme, İstanbul Technical University, Maslak Campus, İstanbul 34469, Turkey; hunlu@fsm.edu.tr; 2 Department of Electrical and Electronics Engineering, Faculty of Engineering, Fatih Sultan Mehmet Waqf University, Topkapı Campus, İstanbul 34015, Turkey

**Keywords:** 2NN *sp*^3^s* and *sp*^3^ tight-binding theories, *k*·*p* effective mass approximation, nanocrystal band gap, CdSe/Cd(Zn)S and ZnSe/Zn(Cd)S core/shell quantum dots

## Abstract

We propose a temperature-dependent optimization procedure for the second-nearest neighbor (2NN) *sp*^3^s* tight-binding (TB) theory parameters to calculate the effects of strain, structure dimensions, and alloy composition on the band structure of heterostructure spherical core/shell quantum dots (QDs). We integrate the thermoelastic theory of solids with the 2NN *sp*^3^s* TB theory to calculate the strain, core and shell dimensions, and composition effects on the band structure of binary/ternary CdSe/Cd(Zn)S and ZnSe/Zn(Cd)S QDs at any temperature. We show that the 2NN *sp*^3^s* TB theory with optimized parameters greatly improves the prediction of the energy dispersion curve at and in the vicinity of L and X symmetry points. We further used the optimized 2NN *sp*^3^s* TB parameters to calculate the strain, core and shell dimensions, and composition effects on the nanocrystal bandgaps of binary/ternary CdSe/Cd(Zn)S and ZnSe/Zn(Cd)S core/shell QDs. We conclude that the 2NN *sp*^3^s* TB theory provides remarkable agreement with the measured nanocrystal bandgaps of CdSe/Cd(Zn)S and ZnSe/Zn(Cd)S QDs and accurately reproduces the energy dispersion curves of the electronic band structure at any temperature. We believe that the proposed optimization procedure makes the 2NN *sp*^3^s* TB theory reliable and accurate in the modeling of core/shell QDs for nanoscale devices.

## 1. Introduction

Since Richard Feynman first proposed the concept of nanotechnology in 1959, scientific research and technological development have accelerated significantly over the past several decades. Increasing speed needs in electronic communications and computer information technologies have significantly accelerated scientific research on the artificial structures made of atoms, molecules, and even biological systems [1]. Semiconductor quantum dots (QDs), being zero-dimensional structures, exhibit a more distinct density of states compared to higher-dimensional systems (e.g., two-dimensional systems), leading to enhanced charge transport and optical properties. Consequently, they have been extensively utilized in the development of diode lasers and transistors.

The physical properties of QDs are strongly influenced by their size and shape. As the size of the crystal decreases to nanometers, the nanocrystal bandgap increases. Quantum dots with a larger size have energy levels that are more closely spaced, enabling them to absorb photons with lower energy, typically those towards the red color end of the UV spectrum. Colloidal core/shell QDs (e.g., Cd(Zn)S/CdSe and Zn(Cd)S/ZnSe) are now designed and produced to protect the active core region from environment. This approach enhances the electrical charge transport and optical luminescence efficiency.

**Figure 1:**
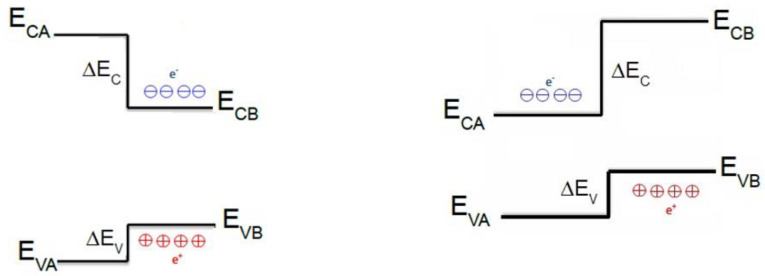
Band diagrams of type I (**left**) and type II (**right**) heterostructures.

When a nanoscale core/shell structure is formed between two dissimilar semiconductors, the band structure exhibits an abrupt change across the interface. As shown in Figure 1, the widegap semiconductor shell overlaps that of the narrow-gap semiconductor core, or the barrier, which can also partially overlap. The potential gradient separates the charge carriers on opposite sides of the interface. The band offsets at the interface cause higher carrier confinement in field-effect transistors and better light emission in optical devices. To describe the behavior of charge carriers (electrons and holes) in QD devices in the presence of internal forces due to strain, as well as external forces due to applied voltages, we need to understand the modifications of the electronic and optical properties across the core/shell interface. Therefore, reliable modeling of the interface band structure is essential for the reliable modeling and precise numerical simulation of charge transport and predicting the performance of QD electronic and optical devices at any temperature.

Theoretical calculations of the semiconductor band structure are often carried out by using the well-known solid-state physics theories: (i) ab initio methods, such as density functional theory [2], allowing one to calculate the electronic structure without using empirical fitting parameters; (ii) empirical methods [3], such as the local/nonlocal empirical pseudopotential method (EPM), orthogonalized plane wave (OPW), *k.p* effective approximation [4,5]; or by semi-empirical tight binding methods [6,7] with various atomic orbital bases (e.g., *sp*^3^, *sp*^3^s*, and *sp*^3^*d*^5^). Although DFT calculations yield satisfactory results for the lattice properties of semiconductors, they give unsatisfying results for bandgaps when compared with experiments. However, the semi-empirical tight-binding theory has appreciable advantages over the density functional theory, since it can be easily implemented in determining electronic properties of core/shell quantum dots.

In this work, we will present a temperature-dependent optimization procedure for determining the tight-binding parameters in the semi-empirical second-nearest neighbor (2NN) *sp*^3^s* TB theory to investigate the effects of interface strain, core and shell dimensions, and composition on the electronic properties of CdSe/Cd(Zn)S and ZnSe/Zn(Cd)S core/shell QDs. We will compare the results with those of the conventional 2NN *sp*^3^ TB theory and *k.p* effective mass approximation. In Section 3, we describe the semi-empirical 2NN *sp*^3^s* tight-binding theory, with the spin–orbit coupling of II cation (Cd, Zn) and VI anion (S, Se) atoms. In Section 4, we discuss the thermoelastic strain modeling in spherical core/shell QDs as a function of device dimensions and alloy composition at any temperature. In Section 5, we will demonstrate that the temperature-dependent optimization procedure makes it possible for the 2NN *sp*^3^s* TB theory to accurately predict measured bandgaps and precisely reproduce the band dispersion curves of group II-VI compounds as a function of the k wave vector. We also show that the 2NN *sp*^3^s* TB theory offers better prediction of the effects of the interface strain, core and shell dimensions, and composition on the bandgaps of CdSe/Cd(Zn)S and ZnSe/Zn(Cd)S heterostructure core/shell QDs at any temperature.

## 2. Semiconductor Band Structure Modeling at Nanoscale

Classical UV–Vis optical absorption spectra indicate a blue shift with the decreasing diameter of a nanocrystal as the first absorption peak energy, which is commonly expressed as

(1) $$E_{g}^{nc}(d)=hc/\lambda_{\max},$$

where *c* is the light speed, *h* is the Planck’s constant, and $\lambda_{\max}$ is the maximum absorption wavelength. Brus [8] explained the spectral shift and calculated the nanocrystal bandgap. In *k.p* effective mass approximation, the Hamiltonian for charged particles is written as [8,9]

(2) $$H=\frac{\hbar^{2}}{2m_{e}^{*}}\nabla^{2}+\frac{\hbar^{2}}{2m_{h}^{*}}\nabla^{2}+V_{e}({\vec{r}}_{e})+V_{h}({\vec{r}}_{h})-\frac{e^{2}}{\varepsilon |{\vec{r}}_{e}-{\vec{r}}_{h}|},$$

where the first and second terms are kinetic energies of electrons and holes, and the third and fourth terms are the confinement potential energies. The last term is the Coulomb potential. 

In the framework of Kane’s *k.p approximation*, the energy states are expanded in a finite set of Bloch states close to an extremum $k_{0}$ of band structure inside the Brillouin zone, where the spin–orbit interaction effect is considered [10]. Assuming strong confinement, the solution of the Schrödinger equation for a particle in a spherical box yields the following expression for the nanocrystal bandgap (first exciton energy level) of a type I core/shell quantum dot [11]

(3) $$E_{g}^{nc}(\varepsilon_{i})=E_{g}^{bi}(\varepsilon_{i})+\frac{2\pi^{2}\hbar^{2}}{m_{cv}^{*}d^{2}}\delta_{sp}\left(1-\frac{2\pi^{2}\hbar^{2}}{m_{cv}^{*}d^{2}}\delta_{sp}\right)-\frac{3.572e^{2}}{\varepsilon_{\infty }d}-\frac{0.124e^{4}}{\hbar^{2}m_{cv}^{*}\varepsilon_{\infty }^{2}},$$

where $E_{g}^{bi}(\varepsilon_{i})$ and $\delta_{sp}=\left(E_{g}^{bi}(\varepsilon_{i})+\Delta_{i}\right)/\left(E_{g}^{bi}(\varepsilon_{i})+2\Delta_{i}/3\right)$ are, respectively, the strain-dependent core bulk bandgap and correction factor. The third term is the Coulomb attraction potential energy, and the fourth term is the Rydberg correlation energy [9]; $\varepsilon_{\infty }$ is the optical dielectric constant of bulk core region. $m_{cv}^{*}=(m_{e}^{*}m_{h}^{*})/(m_{e}^{*}+m_{h}^{*})$ is the reduced effective mass of the electron–hole pair. $m_{e}^{*}$ and $m_{h}^{*}$ are the effective masses of free electrons and holes, respectively. In type II core/shell QDs, the nanoparticle bandgap is written as [11]

(4) $$E_{g}^{nc}(\varepsilon_{i})=E_{g}^{bi}(\varepsilon_{i})-\Delta E_{v}(\varepsilon_{i})+\frac{2\hbar^{2}\pi^{2}}{m_{cv}^{*}d^{2}}\delta_{sp}\left(1-\frac{2\pi^{2}\hbar^{2}}{m_{cv}^{*}d^{2}}\delta_{sp}\right)-\frac{3.572e^{2}}{\varepsilon_{\infty }d}-\frac{0.124e^{4}}{\hbar^{2}m_{cv}^{*}\varepsilon_{\infty }^{2}},$$

where $E_{g}^{bi}(\varepsilon_{i})$ and $\Delta E_{v}(\varepsilon_{i})$ are the strain-dependent core bandgap and the valence-band offset, respectively. In the following section, we will introduce a temperature-dependent optimization procedure for determining the tight-binding parameters in the 2NN *sp*^3^s* TB theory to study the effects of strain, core and shell dimensions, and composition on the electronic properties of CdSe/Cd(Zn)S and ZnSe/Zn(Cd)S QDs.

## 3. Semi-Empirical Second-Nearest Neighbor 2NN *sp*^3^s* TB Theory Modeling

The semi-empirical second-nearest neighbor 2NN *sp*^3^ tight-binding theory yields a good description of the valence-band dispersion curves, but not the conduction-band dispersion curves at the X symmetry point [2]. Vogl et al. [7] added a fictitious excited orbital to mimic the effects of a higher lying d-state to the 2NN *sp*^3^ orbitals set to overcome the inaccuracy of the 2NN *sp*^3^ TB theory. The Schrödinger equation is written in matrix form as

(5) $$\sum_{\beta }\left[H_{\alpha \beta }(k)-S_{\alpha \beta }(k)E\right]=0,$$

where the 2NN *sp*^3^s* Hamiltonian matrix is expressed as

(6) $$H_{\alpha \beta }(k)=\langle \varphi_{\alpha }(k)|H|\varphi_{\beta }(k)\rangle =\varepsilon_{\alpha \beta }+\sum_{i\neq 0}I_{\alpha \beta }(0,i)e^{ik.r_{i}}+H_{so},$$

where $\varepsilon_{\alpha \beta }$ is the on-site energy for the β orbital (*s*, *p*, s*) at the atomic site α (cation and anion) and represents the intra-atomic integrals, which couple atomic orbitals located in the same cell. $I_{\alpha \beta }(0,i)$ are the first-nearest neighbor interaction integrals. $S_{\alpha \beta }=\langle \varphi_{\alpha }(k)|\varphi_{\beta }(k)\rangle$ is the orthogonal overlap integral between the atomic-like orbitals $\left(S_{aa}^{2}+S_{cc}^{2}=1\right)$. Here, $\left|\varphi_{\beta }(k\right)\rangle$ is the basis function formed by a linear combination of s and p orbitals of cation and anion atoms with the wave function coefficient $u_{\beta }$. The Hamiltonian $H_{\alpha \beta }$ matrix consists of thirteen independent matrix elements; six of them are diagonal elements (onsite atomic energies: $\varepsilon_{sa},\varepsilon_{sc},\varepsilon_{pa},\;\varepsilon_{pc},\varepsilon_{s^{*}a},\varepsilon_{s^{*}c}$) and seven of them are off-diagonal elements (interacting integrals, known as hopping terms: $\varepsilon_{ss},\varepsilon_{xx},\varepsilon_{s_{a}p_{c}},\varepsilon_{s_{c}p_{a}},\;\varepsilon_{xy},\varepsilon_{s^{*}p},\varepsilon_{ps^{*}}$). The addition of spin–orbit coupling to the 2NN *sp*^3^s* basis set, in which the spin–orbit interactions for cation and anion atoms are described by two terms: $\lambda_{a}=<x_{a}↑|H_{so}|z_{a}↓>$ and $\lambda_{c}=<x_{c}↑|H_{so}|z_{c}↓>$, respectively, which add two more tight-binding parameters. $\lambda_{a}$ reproduces the bulk zone center, splitting between the split-off band and the light and heavy hole bands. The addition of the 2NN interactions ($\varepsilon_{sx}$ and $\varepsilon_{xy}$) in the *sp*^3^s* basis set adds two extra interaction parameters and increase the size of the (10 × 10) Hamiltonian to (20 × 20) one that is diagonalized for each *k* vector to obtain a band structure [12]. The diagonal and off-diagonal sub-matrices are written as

(7a) $$H_{cc}=\left(\begin{array}{cccccccccc} \varepsilon_{sc} & 0 & -\varepsilon_{sx}B_{6} & 0 & -\varepsilon_{sx}B_{5} & 0 & -\varepsilon_{sx}B_{4} & 0 & 0 & 0\\ 0 & \varepsilon_{sc} & 0 & -\varepsilon_{sx}B_{6} & 0 & -\varepsilon_{sx}B_{5} & 0 & -\varepsilon_{sx}B_{4} & 0 & 0\\ -\varepsilon_{sx}B_{6} & 0 & \varepsilon_{pc} & 0 & -\varepsilon_{xy}B_{4}+i\lambda_{c} & 0 & \varepsilon_{xy}B_{5} & \lambda_{c} & 0 & 0\\ 0 & -\varepsilon_{sx}B_{6} & \varepsilon_{xy}B_{4}+i\lambda_{c} & \varepsilon_{pc} & 0 & \varepsilon_{xy}B_{4}+i\lambda_{c} & -i\lambda_{c} & \varepsilon_{xy}B_{5} & 0 & 0\\ -\varepsilon_{sx}B_{5} & 0 & 0 & 0 & \varepsilon_{pc} & 0 & \varepsilon_{xy}B_{6} & -i\lambda_{c} & 0 & 0\\ 0 & -\varepsilon_{sx}B_{5} & \varepsilon_{xy}B_{5} & \varepsilon_{xy}B_{4}-i\lambda_{c} & 0 & \varepsilon_{pc} & 0 & \varepsilon_{xy}B_{6} & 0 & 0\\ -\varepsilon_{sx}B_{4} & 0 & 0 & -\lambda_{c} & \varepsilon_{xy}B_{6} & iS_{c} & \varepsilon_{pc} & 0 & 0 & 0\\ 0 & -\varepsilon_{sx}B_{4} & \lambda_{c} & \varepsilon_{xy}B_{5} & i\lambda_{c} & \varepsilon_{xy}B_{5} & 0 & \varepsilon_{pc} & 0 & 0\\ 0 & 0 & 0 & 0 & 0 & 0 & 0 & 0 & \varepsilon_{cs^{*}} & 0\\ 0 & 0 & 0 & 0 & 0 & 0 & 0 & 0 & 0 & \varepsilon_{cs^{*}} \end{array}\right),$$

(7b) $$H_{ca}=\left(\begin{array}{cccccccccc} \varepsilon_{ss}B_{0} & 0 & \varepsilon_{sp}B_{1} & 0 & \varepsilon_{sp}B_{2} & 0 & \varepsilon_{sp}B_{3} & 0 & 0 & 0\\ 0 & -\varepsilon_{ss}B_{0} & 0 & -\varepsilon_{sp}B_{1} & 0 & -\varepsilon_{sp}B_{2} & 0 & -\varepsilon_{sp}B_{3} & 0 & 0\\ -\varepsilon_{ps}B_{1} & 0 & \varepsilon_{xx}B_{0} & 0 & \varepsilon_{xy}B_{3} & 0 & \varepsilon_{xy}B_{2} & 0 & -\varepsilon_{ps^{*}}B_{1} & 0\\ 0 & \varepsilon_{ps}B_{1} & 0 & -\varepsilon_{xx}B_{0} & 0 & -\varepsilon_{xy}B_{3} & 0 & \varepsilon_{xy}B_{2} & 0 & \varepsilon_{ps^{*}}B_{1}\\ -\varepsilon_{ps}B_{2} & 0 & \varepsilon_{xy}B_{3} & 0 & \varepsilon_{xx}B_{0} & 0 & \varepsilon_{xy}B_{1} & 0 & \varepsilon_{ps^{*}}B_{2} & 0\\ 0 & \varepsilon_{ps}B_{2} & 0 & -\varepsilon_{xy}B_{3} & 0 & -\varepsilon_{xx}B_{0} & 0 & -\varepsilon_{xy}B_{1} & 0 & \varepsilon_{ps^{*}}B_{2}\\ -\varepsilon_{ps}B_{3} & 0 & -\varepsilon_{xy}B_{2} & 0 & \varepsilon_{xy}B_{1} & 0 & \varepsilon_{xx}B_{0} & 0 & -\varepsilon_{ps^{*}}B_{3} & 0\\ 0 & \varepsilon_{ps}B_{3} & 0 & -\varepsilon_{xy}B_{2} & 0 & -\varepsilon_{xy}B_{1} & 0 & -\varepsilon_{xx}B_{0} & 0 & \varepsilon_{ps^{*}}B_{3}\\ 0 & 0 & \varepsilon_{s^{*}p}B_{1} & 0 & \varepsilon_{s^{*}p}B_{2} & 0 & \varepsilon_{s^{*}p}B_{3} & 0 & 0 & 0\\ 0 & 0 & 0 & -\varepsilon_{s^{*}p}B_{1} & 0 & -\varepsilon_{s^{*}p}B_{2} & 0 & -\varepsilon_{s^{*}p}B_{3} & 0 & 0 \end{array}\right),$$

with similar diagonal and off-diagonal sub-matrices $H_{aa}$ and $H_{ac}=H_{ca}^{*}$. $B_{i}^{*}$ is the complex conjugate of $B_{i}$ representing *k* wave vector dependence, written as

(8a) $$B_{0}(k)=4Cos\left(\frac{k_{x}a}{2}\right)Cos\left(\frac{k_{y}a}{2}\right)Cos\left(\frac{k_{z}a}{2}\right)-4iSin\left(\frac{k_{x}a}{2}\right)Sin\left(\frac{k_{y}a}{2}\right)Sin\left(\frac{k_{z}a}{2}\right),$$

(8b) $$B_{1}(k)=4Cos\left(\frac{k_{x}a}{2}\right)Sin\left(\frac{k_{y}a}{2}\right)Sin\left(\frac{k_{z}a}{2}\right)+4iSin\left(\frac{k_{x}a}{2}\right)Cos\left(\frac{k_{y}a}{2}\right)Cos\left(\frac{k_{z}a}{2}\right),$$

(8c) $$B_{2}(k)=-4Sin\left(\frac{k_{x}a}{2}\right)Cos\left(\frac{k_{y}a}{2}\right)Sin\left(\frac{k_{z}a}{2}\right)+4iSin\left(\frac{k_{x}a}{2}\right)Sin\left(\frac{k_{y}a}{2}\right)Cos\left(\frac{k_{z}a}{2}\right),$$

(8d) $$B_{3}(k)=-4Sin\left(\frac{k_{x}a}{2}\right)Sin\left(\frac{k_{y}a}{2}\right)Cos\left(\frac{k_{z}a}{2}\right)-4iCos\left(\frac{k_{x}a}{2}\right)Cos\left(\frac{k_{y}a}{2}\right)Sin\left(\frac{k_{z}a}{2}\right),$$

(8e) $$B_{4}(k)=4Sin\left(k_{x}a\right)Sin\left(k_{y}a\right),$$

(8f) $$B_{5}(k)=4Sin\left(k_{x}a\right)Sin\left(k_{z}a\right),$$

(8g) $$B_{6}(k)=4Sin\left(k_{y}a\right)Sin\left(k_{z}a\right),$$

where $i=\sqrt{-1}$ and $r_{1}=(a/2)(1,1,1)$, $r_{2}=(a/2)(1,-1,-1)$, $r_{3}=(a/2)(-1,1,-1)$, and $r_{4}=(a/2)(-1,-1,1)$ are the displacement vectors of the nearest neighbor atoms.

The zero-temperature values of diagonal and off-diagonal sub-matrix elements of $H_{\alpha \beta }$ in Equations (6) and (7a,b) are determined by fitting the band gaps to those obtained by the nonlocal pseudopotential theory for bulk semiconductors [3], compared with measured values based on experiments carried out near T = 0 K [13,14]. First, the values of on-site and off-site matrix elements in $H_{\alpha \beta }$ are estimated, followed by a least-squares error minimization procedure at symmetrical points in the energy band dispersion curves to match the band gaps predicted by the nonlocal pseudopotential theory. Lattice misfit-induced strain effects on the off-diagonal elements of the Hamiltonian matrix elements are commonly obtained by using Harrison Scaling rule [15]:

(9) $$V_{ll^{\prime }m}(\varepsilon )=V_{ll^{\prime }m}{\left(a/a_{o}\right)}^{-\eta_{llm}},$$

where $V_{ll^{\prime }m}(\varepsilon )$ and $V_{ll^{\prime }m}$ are the strained and bulk values, respectively. For the hopping interactions between α and β orbitals, the $\eta_{llm}$ exponent is adjusted to reproduce the band structure of semiconductors under hydrostatic pressure, namely the volume deformation potential $\partial E_{g\Gamma }/\partial P,\partial E_{gL}/\partial P,\;$ and the $\partial E_{gX}/\partial P$ of the measured and/or local/nonlocal empirical pseudopotential method (EPM) produced the band gaps $E_{g\Gamma },E_{gL}$, and $E_{gX}$ at high symmetry points, but it is often taken as $\eta_{llm}=2$. In Section 4, we will discuss the effects of the interface strain and composition on the band structure of core/shell QDs.

## 4. Strain and Composition Effect in Core/Shell Quantum Dots

**Figure 2:**
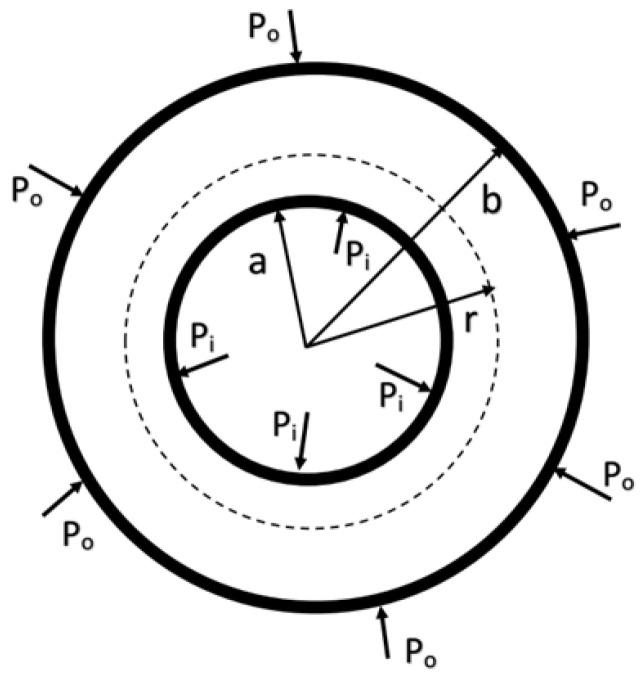
A schematic representation of spherical core/shell QD. The outer part (*a* < *r* < *b*) is defined as shell and the inner part (0 < *r* < *a*) is defined as core.

To model the interface strain in colloidal spherical core/shell QDs, we consider a hollow sphere with inner radius *a* and outer radius *b*, shown in Figure 2. The outer part (*a < r < b*) is defined as the shell and the inner part (*0* < *r* < *a*) is defined as the core, both of which experience inner and outer pressures. Stress–strain relations are written as [16]

(10) $$\varepsilon_{ij}=\frac{1}{E}\left((1+v)\sigma_{ij}-v\sigma_{kk}\delta_{ij}\right)+\alpha \Delta T\delta_{ij},$$

where strain and stress components are $\varepsilon_{ij}$ and $\sigma_{ij}$. *v* and *E* are Poisson ratio and Young modulus. αΔT is the thermal strain developed during crystal growth. $\sigma_{t}=\sigma_{\theta \theta }=\sigma_{\varphi \varphi }$ are stresses and $\varepsilon_{rr}=\varepsilon_{r}$ and $\varepsilon_{\theta \theta }=\varepsilon_{\varphi \varphi }=\varepsilon_{t}$ are the corresponding radial and tangential strains. Because of the spherical symmetry, the shear stresses and strains across the core/shell interface are zero ($\sigma_{r\theta }=\sigma_{r\varphi }=\sigma_{\varphi \theta }=0$ and $\varepsilon_{r\theta }=\varepsilon_{r\varphi }=\varepsilon_{\varphi \theta }=0$). The equilibrium equation for the core/shell structure is written as [16]

(11) $$\;\frac{d\sigma_{r}}{dr}+\frac{2}{r}(\sigma_{r}-\sigma_{t})=0,$$

which is solved by using boundary conditions, namely (i) $\sigma_{ir}(a)=\sigma_{mr}(a)=-P_{i}$, (ii) $\sigma_{mr}(b)=P_{o}=0$, and (iii) the *shrink fit condition*, which is written as

(12) $${\left|r\left(\varepsilon_{m\theta }-\varepsilon_{i\theta }\right)\right|}_{r=a}=a\varepsilon_{im}=a(a_{i}-a_{m})/a_{m},$$

In core region, the solution of Equation (10) yields the expression for the interface pressure $\sigma_{ir}=\sigma_{i\theta }=\sigma_{i\varphi }=\sigma_{i}=-P_{i}$. Equation (10) then gives the expression for strain on the core side

(13) $$\varepsilon_{i}=\frac{(1-2\nu_{i})\sigma_{i}}{E_{i}}+\alpha_{i}T=-\frac{(1-2\nu_{i})P_{i}}{E_{i}}+\alpha_{i}T,$$

where $\left(\varepsilon_{i}=\varepsilon_{ir}=\varepsilon_{i\theta }=\varepsilon_{i\varphi }\right)$. Solving Equation (11) in the shell region, one finds

(14) $$\sigma_{mr}=\frac{a^{3}b^{3}(P_{o}-P_{i})}{(b^{3}-a^{3})r^{3}}+\frac{a^{3}P_{i}-b^{3}P_{o}}{\left(b^{3}-a^{3}\right)},\;\;\;\sigma_{mt}=-\frac{a^{3}b^{3}(P_{o}-P_{i})}{2(b^{3}-a^{3})r^{3}}+\frac{a^{3}P_{i}-b^{3}P_{o}}{\left(b^{3}-a^{3}\right)},$$

Substituting $\sigma_{mr}$ and $\sigma_{mt}$ in Equation (11) at *r = a* with $P_{o}=0$, the following equations are written for the radial and tangential strains on the shell side of the interface

(15a) $$\varepsilon_{mr}=\frac{P_{i}}{E_{m}(b^{3}-a^{3})}[(1-2v_{m})a^{3}-(1+v_{m})b^{3}]+\alpha_{m}T$$

(15b) $$\varepsilon_{mt}=\varepsilon_{m\theta }=\varepsilon_{m\varphi }=\frac{P_{im}}{E_{m}(b^{3}-a^{3})}[(1-2v_{m})a^{3}-(1+v_{m})b^{3}]+\alpha_{m}T,$$

Combining Equation (15a,b) with Equation (13), one finds the interface contact pressure

(16) $$P_{i}=\frac{2E_{i}E_{m}[1-{\left(a/b\right)}^{3}][\varepsilon_{im}+(\alpha_{i}-\alpha_{m})T]}{[(1+v_{m})E_{i}+2(1-2v_{i})E_{m}]+2[(1-2v_{m})E_{i}-(1-2v_{i})E_{m}]{\left(a/b\right)}^{3}},$$

Upon the substitution of Equation (16) into Equation (13), one finds strain acting on the core side. Likewise, upon the substitution of Equation (16) into Equation (15a,b), one finds strains on the shell side.

As one component of the heterostructure QD is a ternary semiconductor, interface strain will be composition dependent. The effect of composition on the lattice structure for ABC ternary in an ABC/AC QD is defined as the combination of the undistorted part ($d_{VCA}=(1-x)d_{AC}^{0}+xd_{BC}^{0}$) and the distorted part ($d_{relax}=x(1-x)\delta_{c}(d_{BC}(x)-d_{AC}(x))$) due to cation–anion relaxation [2], and the composition-dependent ternary bond length is

(17) $$d_{m}(x)=(1-x)d_{AC}(x)+(x)d_{BC}(x)=(1-x)d_{AC}^{0}+xd_{BC}^{0}-x(1-x)\delta_{c}\left(d_{AC}^{0}-d_{BC}^{0}\right),$$

where $d_{AC}(x)$ and $d_{BC}(x)$ are the bond lengths of the *AC* and *BC* binaries of the ABC ternary

(18) $$d_{AC}(x)=d_{AC}^{0}+x\xi_{BC:A}\left(d_{AC}^{0}-d_{BC}^{0}\right);\begin{array}{c} d_{BC}(x)=d_{BC}^{0}+(1-x)\xi_{AC:B}\left(d_{BC}^{0}-d_{AC}^{0}\right), \end{array}$$

where $d_{AC}^{0}$ and $d_{BC}^{0}$ are the undistorted lattice constants of the *AC* and *BC* binaries. $\xi_{AC:B}$ and $\xi_{BC:A}$ are dimensionless coefficients, with the difference $\delta_{c}=\xi_{AC:B}-\xi_{BC:A}$ given as [17]

(19) $$\delta_{c}={\left[1+(\alpha_{AC}+10\beta_{AC})/6\alpha_{BC}\right]}^{-1}-{\left[1+(\alpha_{BC}+10\beta_{BC})/6\alpha_{AC}\right]}^{-1},$$

where α and β are force constants associated with the elastic stiffness constants, and

(20) $$C_{11}+2C_{12}=(3\alpha +\beta )/a-0.355s,\;\;\;C_{11}-C_{12}=4\beta /a+0.053s,$$

where $s=e^{2}Z^{*2}/d^{4}\varepsilon$. As one component of the heterostructure QD is a ternary semiconductor, interface strain will be composition dependent. The composition-dependent lattice mismatch at the interface is $\varepsilon_{im}(x)=(a_{i}-a_{m}(x))/a_{m}(x)$ in ABC/AC ternary/binary core/shell QD. The effects of host and distorted lattice constants by substitutional impurity on the TB parameters of diagonal and off-diagonal submatrices in Equation (7a,b) are [2]

(21a) $$E_{\alpha ,\beta }(x)=(1-x)E_{\alpha ,\beta }(AC)+xE_{\alpha ,\beta }(BC)+x(1-x)\delta_{c}\left(E_{\alpha ,\beta }(AC)-E_{\alpha ,\beta }(BC)\right),$$

(21b) $$d^{2}(x)E_{\alpha ,\beta }(x)=(1-x)E_{\alpha ,\beta }(AC)d_{AC}^{2}+xE_{\alpha ,\beta }(BC)d_{BC}^{2}x(1-x)(d_{AC}^{2}-d_{BC}^{2})\Delta E,$$

where $\Delta E=E_{\alpha ,\beta }(AC)-E_{\alpha ,\beta }(BC)$. $E_{\alpha ,\beta }(AC)$ and $E_{\alpha ,\beta }(BC)$ are the *s*, *p*, and s* atomic energies of *AC* and *BC* binary compounds which form the ABC ternary semiconductor.

**Figure 3:**
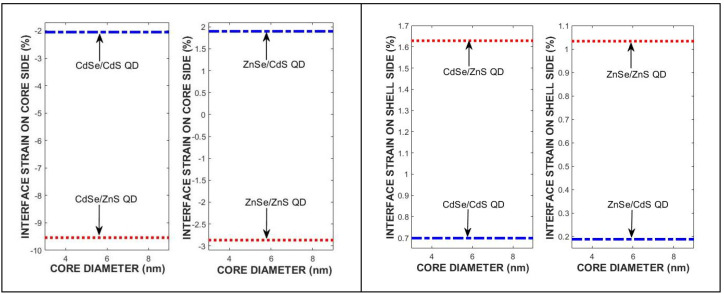
The strain effect on (**left**) the core and (**right**) the shell side of CdSe/Cd(Zn)S and ZnSe/Zn(Cd)S QDs due to core diameter for each quantum dot.

The strain variation with core and shell dimensions in CdSe/Cd(Zn)S and ZnSe/Zn(Cd)S QDs is calculated by using the parameters in Table 1. Figure 3 shows the core diameter effects on the interface strain on the core and shell side for the CdSe/Cd(Zn)S and ZnSe/Zn(Cd)S QDs.

**Figure 4:**
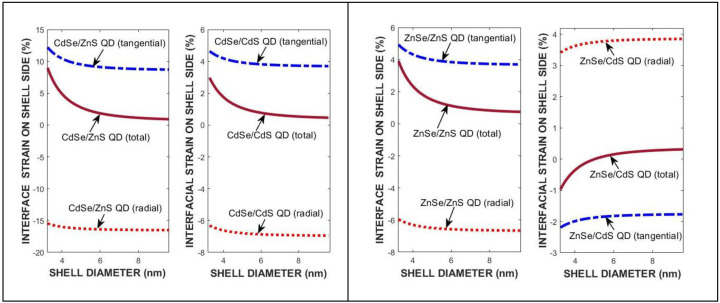
The strain effects in CdSe- (**left**) and ZnSe-(**right**) based QDs with di = 3.0 nm at 300 K.

Figure 4 compares the variation in radial and tangential strain components depending on the shell diameter of QDs.

**Figure 5:**
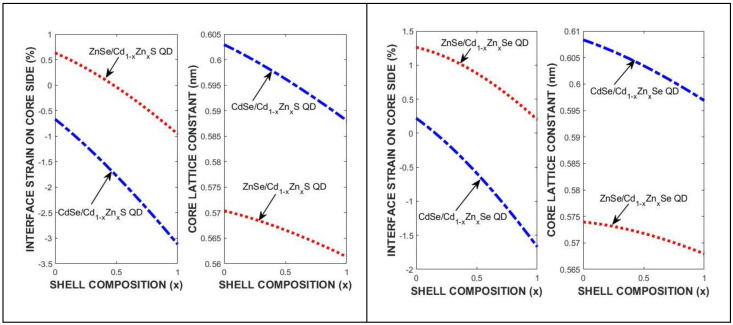
The strain and lattice constant variation with composition in the cores of (**left**) ZnSe/CdZnS and CdSe/CdZnS; and (**right**) ZnSe/CdZnSe and CdSe/CdZnSe QDs for core diameter di = 3.0 nm at 300 K.

The total interface strain is dominated by the tangential component depending on shell diameter in QDs. Figure 5 shows the strain and lattice constant of the core region with composition for various QDs at 300 K. There is a parabolic nonlinear composition effect on the lattice constants of the ternary constituents.

## 5. Results and Discussion

In this section, we will present the results of the band structure calculations carried out by using the optimized 2NN *sp*^3^s* TB parametrization, which are compared with those of the 2NN *sp*^3^ TB theory and *k.p* effective mass approximation for CdSe/Cd(Zn)S and ZnSe/Zn(Cd)S QDs at varying temperatures. We used the material parameters in Table 2 to determine the temperature-dependent optimized parameters in Table 3 for the 2NN *sp*^3^s* tight-binding theory parameters for the bulk CdSe, ZnSe, CdS, and ZnS group II-VI compounds.

**Figure 6:**
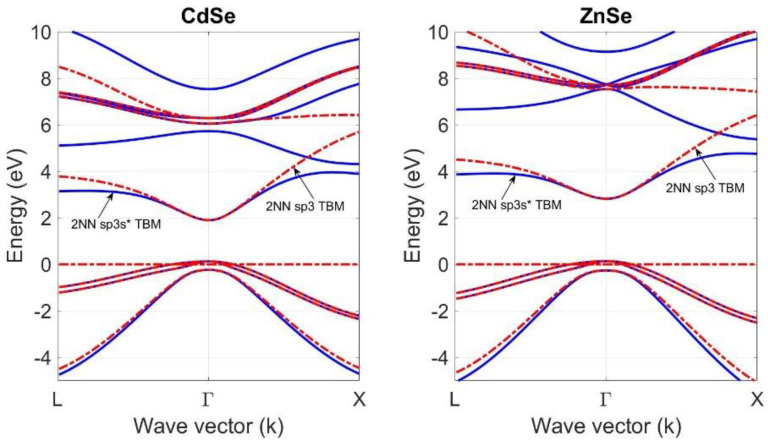
A comparison of the band structure of bulk CdSe and ZnSe compounds at T = 0 K, calculated by using the 2NN *sp*^3^s* TBM and 2NN *sp*^3^ tight-binding theories.

Using the optimized tight-binding parameters in Table 3, the 2NN *sp*^3^s* TB theory is used and compared with the 2NN *sp*^3^ TB theory to calculate the band structure of CdSe and ZnSe compounds at T = 0 K. Figure 6 shows the results of the calculations, which reproduce the conduction- and valence-band structures, including the heavy-hole and light-hole bands and spin–orbit splitting bands.

**Figure 7:**
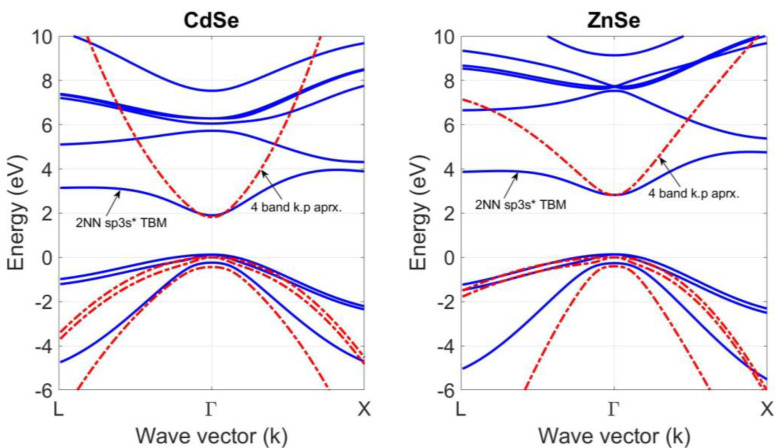
A comparison of the band structure of the bulk CdSe and ZnSe compounds at T = 0 K, calculated by using 2NN *sp*^3^s* TB theory with optimized parameters and *k.p* effective mass approximation.

Although both tight-binding theories accurately reproduce the band structures of these compounds at the Γ high symmetry point, there is noticeable difference between their prediction at and around the L and X symmetry points. As shown in Figure 6, adding a fictitious excited s* state, which mimics the effects of a higher lying d-state, to the *sp*^3^ orbitals set on the cation and anion atoms with 2NN interactions and the spin–orbit coupling of p-states makes the 2NN *sp*^3^s* TB theory greatly improve the simulation of the conduction-band structure of CdSe and ZnSe. This prediction is especially accurate at the X symmetry point. Furthermore, the predictions of 2NN *sp*^3^s* TB theory with optimized parameters in Table 3 are also compared with those of the four-level *k.p* effective mass approximation with material parameters in Table 4. As shown in Figure 7, the predictions of the 2NN *sp*^3^s* TB theory with optimized parameters are in good agreement with those of the four-level *k.p* effective mass approximation at the Γ high symmetry point of the first Brillouin zone of the CdSe and ZnSe compounds at T = 0 K.

Our aim in this work is to use the temperature-dependent optimized tight-binding parameters in the 2NN *sp*^3^s* TB theory to simulate the band structure of constituents of heterostructure core/shell QDs. This study requires the high symmetry point bandgap energies $E_{g\Gamma }(T),E_{gL}(T)\;\mathrm{and}\;E_{gX}(T)$ at high temperatures as input parameters in finding the tight-binding parameters of the 2NN *sp*^3^s* TB theory. We achieve this by using the so-called statistical thermodynamic theory of semiconductors [11], in which bandgap energies at symmetry points of the semiconductors used as core and shell regions are written as

(22) $$E_{gl}^{i}(T)=E_{gl}^{bi}+\Delta C_{ilP}^{0}T(1-\ln T)-\frac{a_{gli}}{B_{i}}\left(P_{i}-\frac{P_{i}^{2}}{2B_{i}}-\frac{\left(1+B_{i}^{\prime }\right)}{3B_{i}^{2}}P_{i}^{3}\right),$$

(23) $$E_{gl}^{m}(T)=E_{gl}^{bm}+\Delta C_{mlP}^{0}T(1-\ln T)-\frac{a_{glm}}{B_{m}}\left(P_{m},-,\frac{P_{m}^{2}}{2B_{m}},-,\frac{\left(1+B_{m}^{\prime }\right)}{3B_{m}^{2}},P_{m}^{3}\right),$$

where $P_{i}(T)=-3B_{i}\varepsilon_{i}(T)=-3B_{i}\alpha \Delta T$ and $P_{m}(T)=-3B\varepsilon_{m}(T)=-3B_{m}\alpha \Delta T$ are the hydrostatic pressures on the core and shell constituents. Here, $a_{gli}$ ($a_{glm}$) is the bandgap deformation potential at Γ, L and X symmetry points and $B_{i}$ ($B_{m}$) is the bulk modulus with $B_{i}^{\prime }=\partial B_{i}/\partial P$ and $B_{m}^{\prime }=\partial B/\partial P$. The second term represents the electron–phonon interactions’ contribution to the bandgap shift. The third term represents the shift in the bandgaps. $C_{cP}^{0}=C_{nP}^{0}-C_{0P}^{0}=C_{pP}^{0}+\Delta C_{P}^{0}$ and $C_{vP}^{0}=C_{pP}^{0}$ are the electron and hole heat capacities; $C_{nP}^{0}=C_{pP}^{0}=(5/2)k$. Here, *k* represents Boltzmann’s constant. $\Delta C_{P}^{0}=C_{nP}^{0}+C_{pP}^{0}-C_{0P}^{0}$ is the heat capacity of the reaction for electron–hole generation:

(24) $$\Delta C_{ilP}^{0}=\frac{1}{T(1-\ln T)}\left(E_{gl}^{ib}(T)-E_{gl}^{bi}(0)+\frac{a_{gli}}{B_{i}}\left(P_{i}-\frac{P_{i}^{2}}{2B_{i}}-\frac{\left(1+B_{i}^{\prime }\right)}{3B_{i}^{2}}P_{i}^{3}\right)\right),$$

(25) $$\Delta C_{mlP}^{0}=\frac{1}{T(1-\ln T)}\left(E_{gl}^{m}(T)-E_{gl}^{bm}(0)+\frac{a_{glm}}{B_{m}}\left(P_{m}-\frac{P_{m}^{2}}{2B_{m}}-\frac{\left(1+B_{m}^{\prime }\right)}{3B_{m}^{2}}P_{m}^{3}\right)\right),$$

where $E_{bi}^{gl}(T)$ and $E_{bm}^{gl}(T)$ are measured bandgaps, which are fitted to [23] as follows:

(26) $$E_{g}(T)=E_{g}(0)-\frac{AT^{2}}{\left(T+B\right)},$$

where *A* and *B* are the fitting constants for bulk semiconductors. In the simulation of $\Delta C_{ilP}^{0}$ and $\Delta C_{mlP}^{0}$ from Equations (24) and (25), one needs to know the temperature-dependent bandgap energies at the Γ, L, and X symmetry points.

**Figure 8:**
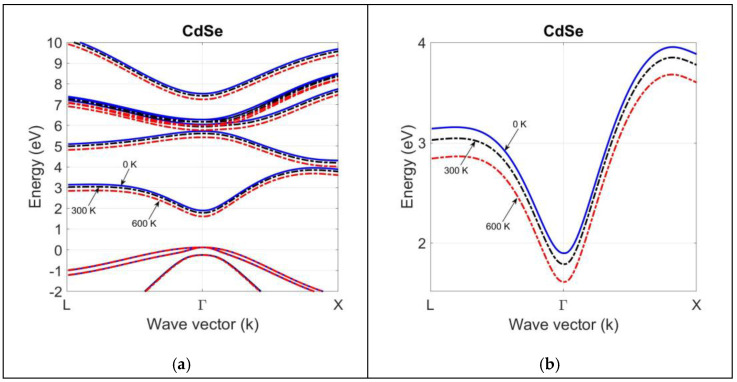
The band structure of bulk CdSe at T = 0 K, 300 K, 600 K, calculated by using the optimized tight-binding parameters in the 2NN *sp*^3^s* TB theory (**a**). A magnified view of the lowest conduction-band structure in (**b**) indicates a larger shift in the bandgap at high symmetry points.

**Figure 9:**
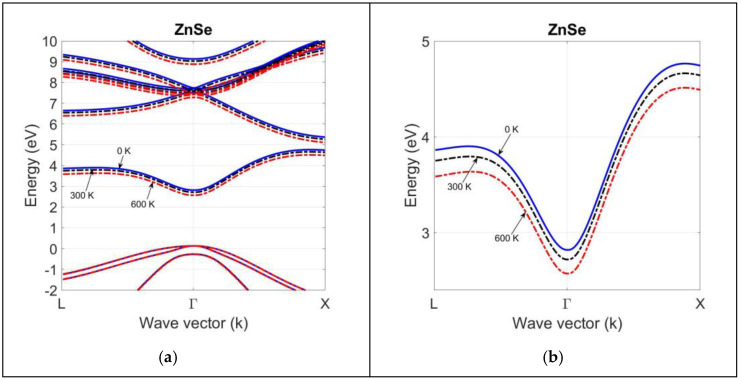
The band structure of bulk ZnSe at T = 0 K, 300 K, 600 K, calculated by using the optimized tight-binding parameters in the frame of 2NN *sp*^3^s* TB theory (**a**). A magnified view of the lowest conduction-band structure in (**b**) indicates a larger shift in the bandgap at high symmetry points.

Since most of the bandgap measurements are carried out for the technologically important direct bandgap energy $E_{g\Gamma }$, we need to make a first-order approximation in finding $\Delta C_{ilP}^{0}$ and $\Delta C_{mlP}^{0}$ for indirect bandgap transitions. At this point, we found it useful to take $\Delta C_{L}^{0}\approx \Delta C_{X}^{0}\approx \Delta C_{\Gamma }^{0}$. Using this approximation and the bandgap deformation potentials $a_{gL}$ and $a_{gX}$ in Table 2, we can find the contribution of electron–phonon interaction to the shift in bandgaps as a function of temperature. Figure 8 and Figure 9 compare the band structure of CdSe and ZnSe compounds at T = 0 K, 300 K, and 600 K. They are calculated by using the 2NN *sp*^3^s* TB theory, with optimized parameters.

Since the deformation potential of the average valence-band edge is small (Table 2), there is not much temperature shift in the valence-band dispersion curve for both compounds. However, the bandgap deformation potentials of group II-VI compounds are large, and a shift in the conduction-band dispersion curve occurs with temperatures at high symmetry points. This is clearly seen in the magnified views of the conduction-band dispersion curves for CdSe and ZnSe compounds. As shown in the magnified views of CdSe and ZnSe band structures, the rise in temperature causes a decrease in the bandgap energies at high symmetry points, as well as in the entire band structure below their 0 K values.

The electron–phonon interaction and thermal strain effects have a greater impact on the temperature variation in the conduction-band structure than the valence-band structure, since the deformation potentials of conduction-band edges are larger than those of average valence-band edge. Table 5 compares the high-symmetry-point bandgap predictions of 2NN *sp*^3^s* TB and 2NN *sp*^3^ TB theory against the available experimental data. The 2NN *sp*^3^s* TB performs better than the 2NN *sp*^3^ TB theory, proving the need for adding the fictitious excited s* state to the *sp*^3^ orbitals set on the cation and anion atoms with 2NN interactions and the spin–orbit coupling of p-states to improve the simulation of the conduction-band structure of II-VI compounds, especially at the X symmetry point at any temperature.

**Figure 10:**
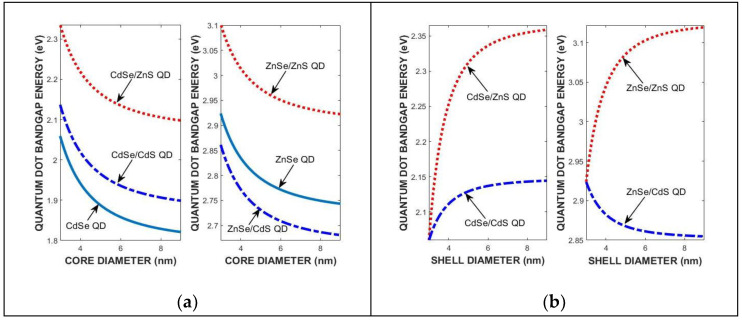
The core (**a**) and shell (**b**) diameter variations in the nanocrystal bandgap energies of four QDs, determined from Equations (3) and (4) at 300 K.

As an extension of the 2NN *sp*^3^s* TB theory and *k.p* effective mass approximation (EMA), Figure 10 illustrates the effects of core- and shell diameter-dependent strain, quantum confinement, and electron–hole Coulomb interactions on the nanocrystal bandgap in four binary/binary heterostructure QDs at 300 K. In these calculations, the optimized tight-binding parameters in Table 3 are used to determine the 0 K band structure of core/shell constituents in the frame of the 2NN *sp*^3^s* TB theory, and the effect of interface strain, core and shell dimensions, quantum confinement, and electron–hole Coulomb interactions, and correlation energy are added through Equations (3) and (4) for type I and type II heterointerface band alignments, respectively.

Table 6 compares the predicted and UV–Vis spectrometer-measured nanocrystal bandgaps of CdSe/ZnS, CdSe/CdS, ZnSe/ZnS, and ZnSe/CdS QDs at 300 K for core diameter d_i_ = 3 nm and varying shell diameter d_m_ = 1.5d_i_. The 0K band structure of core/shell constituents are calculated by using the 2NN *sp*^3^s* and 2NN *sp*^3^ TB theories, and the effect of interface strain, core and shell dimensions, quantum confinement, and electron–hole Coulomb interaction energies are added through Equations (3) and (4) for type I and type II heterointerfaces.

It is a well-known fact that semiconductor alloys (ternary or quaternary) improve the performance of small and nanoscale heterostructure devices, because they allow the device designer to locally modify the band structure of the semiconductor (e.g., increasing direct bandgap) and in turn control the motion of the charge carriers. As one of the constituents of the spherical core/shell QD is a ternary semiconductor alloy (e.g., CdSe/CdZnS, ZnSe/ZnCdS), the alloy composition of the ternary constituent can influence interface strain and in turn the nanocrystal bandgap of the spherical binary/ternary core/shell QDs. To understand how such local modification of band structure can affect the motion of charge carriers, one needs to understand the composition variation in the valence- and conduction-band structure of alloy constituent semiconductors as a function of wave vector.

**Figure 11:**
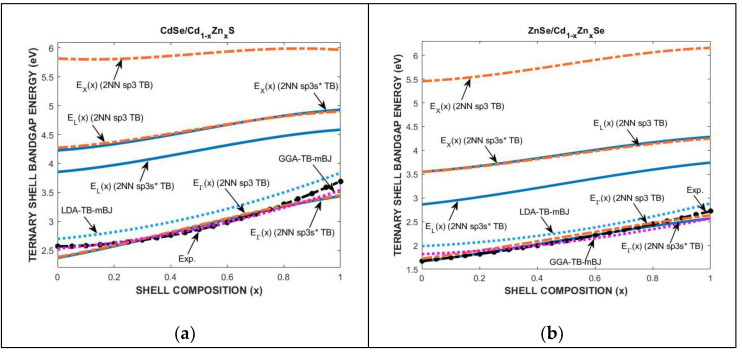
The composition effect on the bandgap energies at the Γ, L, and X symmetry points for (**a**) CdSe/CdZnS and (**b**) ZnSe/CdZnSe, QDs with di = 3.0 nm at T = 300 K.

We used the 2NN *sp*^3^ and 2NN *sp*^3^s* TB theories to investigate the effect of strain, core and shell dimensions, and ternary alloy composition on the electronic band structures of the CdZnS and CdZnSe constituents of CdSe/CdZnS, ZnSe/CdZnSe core/shell QDs in k-space. The effect of ternary composition on the optimized tight-binding parameters is calculated by implementing the modified virtual crystal approximation in the 2NN *sp*^3^ and 2NN *sp*^3^s* TB theories. Figure 11a,b show the composition variation in the bandgaps at the Γ, L, and X high symmetry points of the first Brillouin zone of the energy dispersion curves of CdZnS and CdZnSe ternaries in the CdSe/CdZnS, ZnSe/CdZnSe heterostructures. Figure 11a,b also compare the predictions of the 2NN *sp*^3^ and 2NN *sp*^3^s* TB theories with the first principle’s density functional theory (DFT), demonstrating the variation in the direct and indirect bandgaps’ shell bandgap energy of (a) CdSe/Cd_1-x_Zn_x_S and (b) ZnSe/Cd_1-x_Zn_x_Se core/shell QDs. The calculations are also compared with the density functional theory of Mimouni et al. [24] and the measured bandgaps of A. John Peter and C.W. Lee [25] and N. Samarth et al. [26]. The accuracy of the conduction-band energy levels at the X and L symmetry points indicates that the 2NN *sp*^3^s* TB theory is a good technique for the band structure modeling of semiconductors at high temperatures.

The overall results suggest that the optimized 2NN *sp*^3^s* TB parametrization enhances the accurate prediction of the high-symmetry-point bandgap energies and reproduces the energy band dispersion curves of the binary and ternary constituents of heterostructure spherical core/shell quantum dots at any temperature. The electron–phonon interaction and thermal strain effects are shown to have appreciable effects on the temperature variation in the conduction-band structure than valence-band structure, since the deformation potentials of conduction-band edges are larger than those of the average valence-band edge. There is good agreement between the predictions of the 2NN *sp*^3^s* TB theory and the density functional GGA-TB-mBJ theory. However, DFT calculations are computationally intensive and cannot be easily implemented for nanoscale devices. We should also add that, just as with any other theories, the accuracy of the 2NN *sp*^3^s* TB theory relies on the input parameters being based on a precise description of the band structures of II-VI compound semiconductors by the conventional non-local pseudopotential theory and experimental data. The semi-empirical 2NN *sp*^3^s* TB theory has appreciable advantages over the density functional GGA-TB-mBJ theory, since it can be easily implemented in determining the electronic band structures of the core/shell quantum dots. We aim to explore the proposed optimization procedure for the predictions of the tight-binding parameters applied to new material structures, such as AgBiS2 quantum dot solar cells [27]. We believe that the proposed optimization procedure allows the 2NN *sp*^3^s* TB theory to be easily and effectively implemented in the modeling and simulation of QDs for designing nanoscale devices operating at high temperatures.

## 6. Conclusions

We presented a temperature-dependent optimization procedure for semi-empirical second-nearest neighbor (2NN) *sp*^3^s* tight-binding (TB) theory parameters to calculate the effects of strain, structure dimensions, and alloy composition on the band structure of spherical heterostructure core/shell quantum dots (QDs). We integrated the thermoelastic theory of solids with the 2NN *sp*^3^s* TB theory to calculate the strain, core and shell dimensions, and ternary composition effects on the band structure of binary/ternary CdSe/Cd(Zn)S and ZnSe/Zn(Cd)S heterostructure spherical core/shell QDs at any temperature. There is excellent agreement between the three models at and in the vicinity of the Γ symmetry point of the first Brillouin zone of these compounds. We found that, with the temperature-dependent optimization of tight-binding parameters, the 2NN *sp*^3^s* TB theory greatly improves the prediction of the energy dispersion curve at and in the vicinity of the L and X symmetry points. We conclude that the predictions of the 2NN *sp*^3^s* TB theory, with optimized parameters, provides remarkable agreement with the measured nanocrystal bandgaps of CdSe/Cd(Zn)S and ZnSe/Zn(Cd)S core/shell QDs and accurately reproduces the energy dispersion curves of the electronic band structure at any temperature. We believe that the proposed temperature-dependent optimization procedure makes the semi-empirical 2NN *sp*^3^s* TB theory qualitatively reliable and quantitatively accurate in the modeling of the electronic properties of core/shell QDs for the simulation of nanoscale devices at any temperature.

## Figures and Tables

**Table 1:** Material parameters used in 2NN *sp*^3^s* TB theory and *k.p* approximation [11,13].

Parameter	CdSe	ZnSe	CdS	ZnS
a (nm)	0.607	0.5668	0.581	0.541
C_11_ (10^11^ dyn/cm^2^)	6.67	8.57	7.70	10.11
C_12_ (10^11^ dyn/cm^2^)	4.63	5.07	5.39	6.46
C_44_ (10^11^ dyn/cm^2^)	2.23	4.05	2.36	4.46
$\alpha_{th}$(10^−6^ K^−1^)	7.30	7.60	4.05	6.9

**Table 2:** Bandgaps of some compounds at high symmetry points at 0 K [13,18].

Parameters (eV)	CdSe	ZnSe	CdS	ZnS
$E_{g\Gamma }$	1.899	2.824	2.503	3.702
$E_{gL}$	3.097	3.999	3.983	4.810
$E_{gX}$	3.784	4.54	4.341	5.103
$a_{g\Gamma }$	−2.89	−5.1	−2.9	−5.2
$a_{gL}$	−1.17	−1.74	−1.38	−1.97
$a_{gX}$	1.81	2.16	1.62	2.1

**Table 3:** Optimized 2NN *sp*^3^s* parameters (in eV) for CdSe, CdS, ZnSe, the ZnS at T = 0 K.

**Parameters (eV)**	**CdSe**	**ZnSe**	**CdS**	**ZnS**
$\varepsilon_{sa}$	−9.6269	−11.6988	−11.5323	−11.6051
$\varepsilon_{pa}$	1.4732	1.6485	0.5276	1.4850
$\varepsilon_{sc}$	0.0308	0.0174	1.8316	1.1078
$\varepsilon_{pc}$	4.7309	5.9944	5.8716	6.5178
$\varepsilon_{s^{*}a}$	7.5313	7.7404	7.1313	8.0799
$\varepsilon_{s^{*}c}$	5.7214	9.1361	6.8713	8.0199
4V_s,s_	−4.6402	−6.3791	−3.07	−6.3016
4V_x,x_	2.6399	3.1425	1.7602	3.1111
4V_x,y_	5.3597	6.0971	4.2308	5.0002
4V_s,p_	4.5705	3.8406	2.1695	5.1633
4V_p,s_	5.5392	6.3983	5.4814	5.1685
4V_s*,p_	3.05	2.5995	1.99	2.8902
4V_p,s*_	2.4897	3.9394	3.0605	1.7495
$\varepsilon_{sx}$	0.0	−0.15	0.10	0.20
$\varepsilon_{xy}$	0.0	0.60	−0.01	−0.15
$\lambda_{a}$	0.14	0.16	0.03	0.03
$\lambda_{c}$	0.06	0.03	0.01	0.03

**Table 4:** Parameters used for *k.p* effective mass approximation calculations.

Parameters	CdSe	ZnSe	CdS	ZnS
a (nm)	0.607 ^a^	0.5668 ^a^	0.581 ^a^	0.541 ^a^
$E_{g\Gamma }$ (eV)	1.823	2.823	2.552	3.820
−E_v_ (eV)	11.49	12.65	12.61	14.66
Δ (eV)	0.410 ^a^	0.424 ^a^	0.070 ^a^	0.092 ^a^
a_g_ (eV)	−2.9 ^b^	−5.82 ^c^	−2.94 ^b^	−6.4 ^c^
a_v_ (eV)	0.9 ^b^	1.65 ^c^	0.40 ^b^	2.31 ^c^
$\gamma_{1}$	3.33 ^d^	3.77 ^e^	4.11 ^f^	2.54 ^e^
$\gamma_{2}$	1.11 ^d^	1.24 ^e^	0.77 ^f^	0.75 ^e^
$\gamma_{3}$	1.11 ^d^	1.67 ^e^	1.53 ^f^	1.09 ^e^

^a^ Ref. [13]; ^b^ Ref. [19]; ^c^ Ref. [20]; ^d^ Ref. [21]; ^e^ Ref. [22]; ^f^ Ref. [14].

**Table 5:** Comparison of high-symmetry-point bandgaps of CdSe and ZnSe compounds at 300 K calculated by 2NN *sp*^3^s* and 2NN *sp*^3^ TB theories with experimental (Exp) data or estimated (Est).

Bandgap (eV)	CdSe			ZnSe		
	*sp*^3^s*	*sp* ^3^	Exp.	*sp*^3^s*	*sp* ^3^	Exp.
$E_{g\Gamma }$	1.791	1.851	1.732 [13]	2.721	2.787	2.72 [14]
$E_{gL}$	2.989	3.664		3.887	4.391	3.8 [14]
$E_{gX}$	3.676	5.586	4.37 (Est [14])	4.437	6.315	3.4 [14]

**Table 6:** Comparison of calculated nanocrystal bandgaps of CdSe/ZnS, CdSe/CdS, ZnSe/ZnS, and ZnSe/CdS QDs with experimental data [11] at 300 K. First and second rows indicate the 2NN *sp*^3^s* and 2NN *sp*^3^ TB theories, respectively, for core diameter d_i_ = 3 nm and shell diameter d_m_ = 1.5d_i_.

Core/Shell QD	Calculated Bandgap (eV)	Measured Band Gap (eV)
ZnSe/ZnS	3.070	3.080
	3.136	
ZnSe/CdS	2.873	2.850
	2.939	
CdSe/ZnS	2.289	2.255
	2.349	
CdSe/CdS	2.122	2.309
	2.182	

## Data Availability

The original contributions presented in this study are included in the article. Further inquiries can be directed to the corresponding author.
